# Typhoid Outbreak in Songkhla, Thailand 2009–2011: Clinical Outcomes, Susceptibility Patterns, and Reliability of Serology Tests

**DOI:** 10.1371/journal.pone.0111768

**Published:** 2014-11-06

**Authors:** Wannee Limpitikul, Narong Henpraserttae, Rachanee Saksawad, Kamolwish Laoprasopwattana

**Affiliations:** 1 Department of Pediatrics, Songkhla Hospital, Songkhla, Thailand; 2 Bureau of Epidemiology, Department of Disease Control, Ministry of Public Health in Songkhla, Songkhla, Thailand; 3 Department of Pediatrics, Hat Yai Education Center, Hat Yai Hospital, Hat Yai, Songkhla, Thailand; 4 Department of Pediatrics, Faculty of Medicine, Prince of Songkla University, Hat Yai, Songkhla, Thailand; University Medical Center Groningen, Netherlands

## Abstract

**Objective:**

To determine the clinical manifestations and outcomes, the reliability of *Salmonella enterica* serotype Typhi (*S* ser. Typhi) IgM and IgG rapid tests, and the susceptibility patterns and the response to treatment during the 2009–2011 typhoid outbreak in Songkhla province in Thailand.

**Method:**

The medical records of children aged <15 years with *S* ser. Typhi bacteremia were analysed. The efficacy of the typhoid IgM and IgG rapid tests and susceptibility of the *S* ser. Typhi to the current main antibiotics used for typhoid (amoxicillin, ampicillin, cefotaxime, ceftriaxone, co-trimoxazole, and ciprofloxacin), were evaluated.

**Results:**

*S* ser. Typhi bacteremia was found in 368 patients, and all isolated strains were susceptible to all 6 antimicrobials tested. Most of the patients were treated with ciprofloxacin for 7–14 days. The median time (IQR) of fever before treatment and duration of fever after treatment were 5 (4, 7) days and 4 (3, 5) days, respectively. Complications of ascites, lower respiratory symptoms, anemia (Hct <30%), and ileal perforation were found in 7, 7, 22, and 1 patients, respectively. None of the patients had recurrent infection or died. The sensitivities of the typhoid IgM and IgG tests were 58.3% and 25.6% respectively, and specificities were 74.1% and 50.5%, respectively.

**Conclusion:**

Most of the patients were diagnosed at an early stage and treated with a good outcome. All *S* ser. Typhi strains were susceptible to standard first line antibiotic typhoid treatment. The typhoid IgM and IgG rapid tests had low sensitivity and moderate specificity.

## Introduction

Enteric fever (typhoid or paratyphoid fever) is an important cause of morbidity in developing countries, particularly among children and adolescents in Africa and South and Southeast Asia. The incidence in some counties is as high as 800/100,000 people/year. In 2000, the estimated number of enteric fever cases worldwide was 26.9 million, with 200,000 deaths [1].

The mortality rate in any particular country or area varies according to several factors, such as early diagnosis or available treatment. In patients treated prior to developing a serious complication such as bowel perforation the mortality rate is lower than 1%, but in patients treated only after developing a serious complication the mortality rate is as high as 15% [2]–[4].

In Thailand, where enteric fever is endemic, the incidence varies according to region (central, northern, north-eastern, and southern), and whether or not there is a current outbreak. For example, in 2013 the incidence among the different regions of Thailand varied from 0.9 to 83.6/100,000 people [5], while during 2009–2011, during a typhoid fever outbreak in Songkhla province in the south of Thailand, the estimated incidence increased from 1.8 (the average incidence in the 5 years before the outbreak) to 25.2/100,000 people [6].

Blood culture is the standard method for confirming a typhoid diagnosis; however this method is problematic for resource limited areas, and there is also a time problem, as cultures need at least 1–2 days to report the results, and effective treatment must begin as soon as possible, so a 2-day delay while waiting for a positive diagnosis is not good for the patients. In addition, the sensitivity of blood culture varies in its reliability - if performed during the first seven days of fever it is 60–80%, but if blood is cultured after the 7^th^ day of fever the sensitivity drops to 20–30% [7], [8]. Also, blood cultures can be influenced by previously received antibiotics and/or the volume of blood which need blood to broth ratio of 1∶5 to 1∶10 [9]. The currently most commonly used serology test, the Widal test, has low specificity to diagnose typhoid fever and reports on other recently developed rapid tests for enteric fever have found varying results for the sensitivity and specificity of these tests [10]–[21].

A developing problem in dealing with various diseases including typhoid fever is that in recent years multidrug-resistant bacteria have been increasingly encountered. In South and Southeast Asia, *Salmonella enterica* serotype Typhi (*S* ser. Typhi) resistance to the current first-line treatment, fluoroquinolones, is increasing [22]–[26]. Close monitoring of *S* ser. Typhi resistance to first line antibiotics, and knowing the resistance profile of *S* ser. Typhi, is important in helping the physician to choose the appropriate antibiotic when an outbreak occurs.

This study aimed to determine the clinical manifestations and outcomes, the reliability of *S* ser. Typhi IgM and IgG rapid tests, and the susceptibility patterns and the response to treatment during the 2009–2011 typhoid outbreak in Songkhla province in Thailand.

## Materials and Methods

Permission from the institutional review board of Prince of Songkla University was obtained prior to conducting the study. We retrospectively reviewed the medical records of all children (<15 years of age) from 3 major hospitals in Songkhla province, Thailand (Songkhla Hospital, Songklanagarind Hospital and Hat Yai Hospital) treated during the October 2009–July 2011 typhoid outbreak in this area. Our study involved the use of patient medical data, from which any information that could specifically identify any patient was removed before the analysis was performed. All patients were diagnosed for typhoid fever through a blood culture positive for *S* ser. Typhi, using a BD Phoenix Automated Microbiology System (Becton Dickinson, Sparks, MD). Blood cultures were performed by technicians in the hospitals where the patients first presented.

Demographic characteristics, clinical profiles, treatments and complications, and typhoid immunochromatographic assays for IgM and IgG (SD Bioline *S* ser. Typhi IgG/IgM Test, South Korea) performed on the same day as the blood cultures, were recorded. The *S* ser. Typhi IgG/IgM rapid tests (available only in patients who visited Songkhla Hospital) were immediately performed after the blood samples were collected and the results were interpreted within 15–30 minutes by one of the technicians in Songkhla Hospital. Blood cultures were used as the gold standard to determine the sensitivity, specificity, negative predictive value (NPV), and positive predictive value (PPV) of the SD Bioline *S* ser. Typhi IgG/IgM tests in patients suspected of typhoid fever who lived in the outbreak area with febrile illness without another cause identified. Antimicrobial susceptibility testing (BBL Sensi-Disc; Becton Dickinson, USA) was performed using the Clinical and Laboratory Standards Institute (CLSI) standard disk diffusion method [27]. Six commonly used antimicrobial agents were evaluated: amoxicillin, ampicillin, cefotaxime, ceftriaxone, co-trimoxazole, and ciprofloxacin. Pulsed field gel electrophoresis (PFGE) was performed using the method of Ward et al. [28].

### Statistical analysis

Data were evaluated using descriptive statistics (mean and standard deviation, median and interquartile range (IQR), or frequency and percentage, as appropriate). Comparisons between groups of patients were made using the Student's *t*-test or Mann-Whitney *U-*test for normally distributed and non-normally distributed continuous variables, respectively. Chi-square or Fisher's exact test was used for comparisons of categorical data. R 3.0.2 for Windows was used for statistical analysis.

## Results

### Outbreak periods and patients

**Figure 1:**
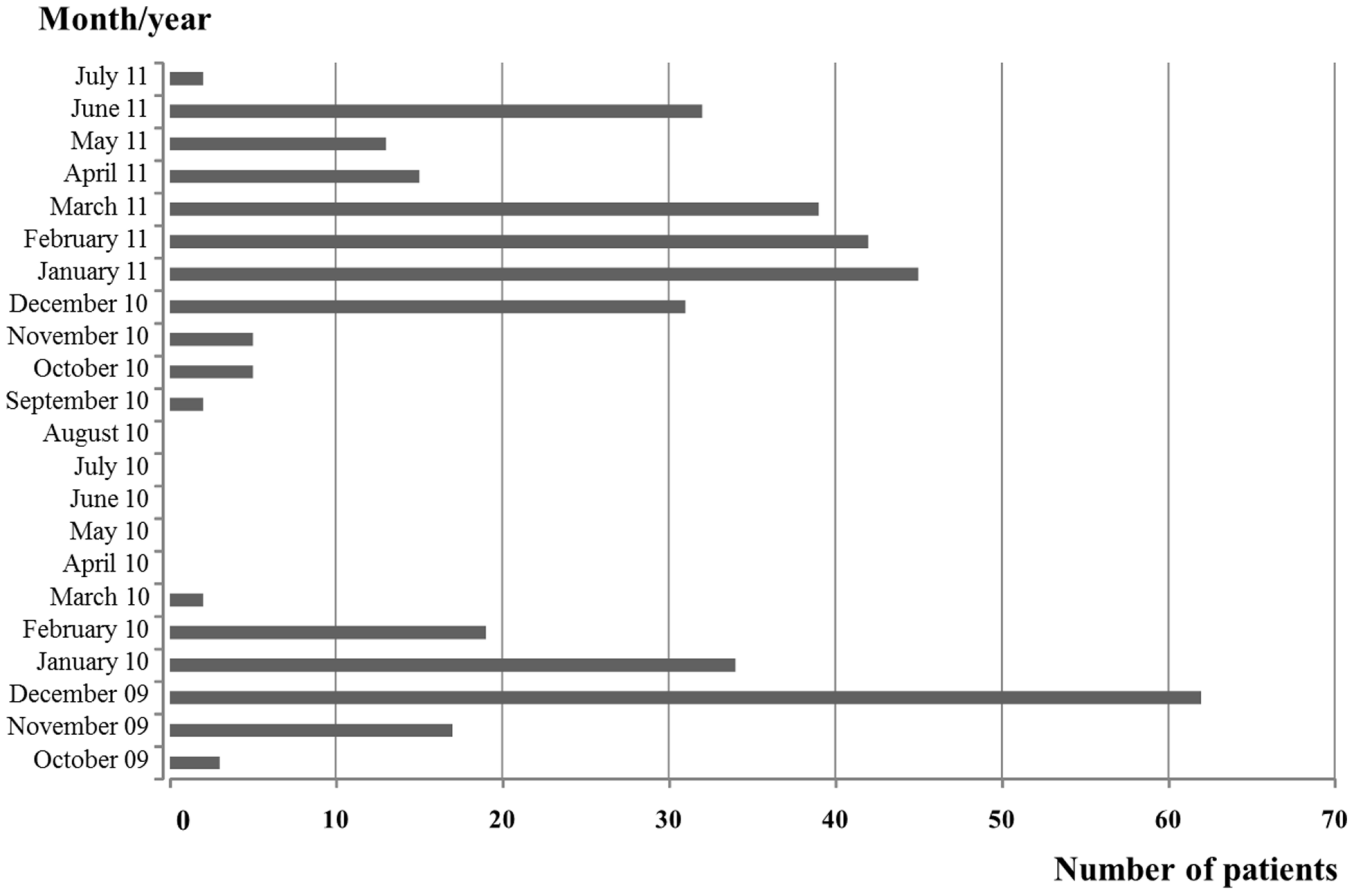
The first and second periods of the 2009–2011 typhoid fever outbreak, showing cases by month and hospital.

*S* ser. Typhi bacteremia was found in 333, 27, and 8 patients from Songkhla Hospital, Songklanagarind Hospital and Hat Yai Hospital, respectively, for a total of 368 cases. Most of the patients were students from at least 10 primary schools in Songkhla province. The mean age of all patients was 7.5±3.0 years, and 44.8% were male. There were 2 separate outbreaks during the Songkhla epidemic, the 1^st^ from October 2009 to March 2010 and the 2^nd^ from September 2010 to July 2011 (Figure 1), with 137 (37.2%) and 231 (62.8%) patients in the two periods, respectively. The clinical characteristics, mean day of fever when initial treatment was begun (5.1±2.7 vs 5.5±2.9 days, p = 0.18) and outcomes were not different between the 1^st^ and 2^nd^ periods of the outbreak. However, the mean age of the patients in the1^st^ period was greater than in the 2^nd^ period (8.0±2.8 vs 7.2±3.0 years, p = 0.01). All of the *S* ser. typhi strains isolated in both periods were susceptible to all six of the tested antibiotics.

During the peak of the 1^st^ outbreak (December 2009–January 2010), the Songkhla branch of the Bureau of Epidemiology, Department of Disease Control, Thailand Ministry of Public Health found that residual chlorine in the water supply from the schools with the highest numbers of typhoid cases schools was 0.1 parts per million (ppm), with no *S* ser. Typhi strains isolated from the tap water in either school, and only non-Typhi *Salmonella* strains found from rectal swabs in 16/88 (18%) of food handlers (4/7, 1/19, and 11/62 food handlers from schools A and B and street venders in front of schools A and B). All 28 food and water samples from these schools were organism-free. However, in market A, non-Typhi *Salmonella* was found in raw pork and raw chicken and *Aeromonas caviae* was found in coconut milk being sold. After 14 days of ciprofloxacin, these 4 and 18 food handlers form schools A and B had a repeat rectal swab, and no growths were found. PFGE from 52 samples (different schools and month of outbreak) had the same pattern of 92–100%. During the second outbreak, the Ministry of Public Health in Songkhla tried to find the source of *S* ser. Typhi as in the first outbreak, but none of samples collected form the environment or food handlers rectal swabs found *S* ser. Typhi.

### Clinical characteristics and outcomes of typhoid patients treated before and after 5 days of fever

All of the 368 confirmed typhoid patients initially presented with high grade fever. Non-mucus bloody diarrhea, vomiting, abdominal pain, and headache were found in 48.4%, 36.7%, 22.8%, and 21.2% of the patients, respectively. The hospitalization rate was 70.7% and the mean duration of hospitalization was 4.7±2.8 days. Most of the hospitalized patients were initially treated with intravenous ceftriaxone or ciprofloxacin, and then continued with oral ciprofloxacin when their clinical status improved and they could tolerate oral intake for the treatment duration of 10–14 days. Most of the out-patients were treated with oral ciprofloxacin for 7–14 days. Sixteen patients were treated with other antibiotics - oral amoxicillin/clavulanate, co-trimoxazole, or azithromycin were given to those who could not tolerate ciprofloxacin (7, 5 and 4 patients, respectively). None of the patients had recurrent infection and all survived.

Of the 357 patients who self-reported the first date of fever, all except 3 were treated within 14 days of the onset of fever. The median day (IQR) of fever before treatment was 5 (4, 7) days and duration of fever after treatment was 4 (3, 5) days (267 patients had available last date of fever data). Patients whose treatment began after day 5 of their fever were more likely to have mucus bloody diarrhea, hepatomegaly, hospitalization, and anemia than those who were treated on or before the 5^th^ day (Table 1, 2).

**Table 1:** Clinical characteristics and outcomes of typhoid patients treated before and after the 5^th^ day of fever.

Clinical factors and outcomes	Stage of fever when treatment begun		P
	≤5 days N = 208	>5 days N = 149	
**Clinical characteristics**			
BT, °C, mean ± SD	40.2±0.7, n = 138	40.0±0.7, n = 116	0.35
Pulse rate/minute, mean ± SD	126±11, n = 133	122±15, n = 111	0.04
Diarrhea, no. (%)	94 (45.2)	83 (55.7)	0.06
Mucus bloody, no. (%)	0	5 (3.4)	0.01
Abdominal pain, no. (%)	52 (25.0)	32 (21.5)	0.52
Vomiting, no. (%)	86 (41.3)	49 (32.9)	0.13
Cough, no. (%)	68 (32.7)	57 (38.3)	0.33
Hepatomegaly, no. (%)	10 (4.8)	20 (13.4)	<0.01
Splenomegaly, no. (%)	0	3 (2.0)	0.07
**Outcomes**			
Hospitalization, no. (%)	141 (67.8)	119 (79.9)	0.01
Complication, total, no. (%)	23 (11.1)	25 (16.8)	0.12
GI complication, no. (%)	0	8	<0.01
Non-GI complication, no. (%)	23	17	0.91

BT, body temperature; GI, gastrointestinal tract.

**Table 2:** Comparison of complete blood counts results tested before and after the 5^th^ day of fever.

Laboratory investigation	Period of fever when tested		p
	≤5 days	>5days	
	N = 221	N = 128	
Hematocrit, %, median (IQR)	35.7±3.8	34.3±4.0	<0.01
Hematocrit <35%, n, (%)	79 (35.7)	63 (49.2)	0.01
Hematocrit <30%, n, (%)	11 (5.0)	13 (10.2)	0.07
White blood cells, cells/mm^3^, median (IQR)	6,800	6,800	0.53
	(5,400–8,710)	(5,300–8,075)	
Neutrophils, %, mean ± SD	60.7±13.0	58.4±12.2, n = 127	0.10
Lymphocytes, %, mean ± SD	29.4±11.9, n = 158	33.3±11.2, n = 104	<0.01
Platelets x 10^3^/mm^3^, median (IQR)	198 (159–245)	200 (160–253)	0.61
	n = 212	n = 115	
<100,000 platelets/mm^3^, n, (%)	11 (5.2)	4 (3.5)	0.59

Complications, mainly moderate to severe gastrointestinal (GI) symptoms, lower respiratory symptoms, anemia (Hct <30%), thrombocytopenia (<100,000 platelets/mm^3^), seizure, and acute kidney injury, were found in 49/368 (13.3%) patients; 5 patients had 2 complications and none died. Complications associated with moderate to severe GI symptoms were found in 8 patients; ascites with abdominal pain (confirmed by abdominal ultrasonography) was found in 7 patients and 1 patient who had fever 28 days prior to treatment had a terminal ileal perforation. None of the patients who were treated prior to the 5^th^ day of fever had GI complications (Tables 1 and 2). Of the 7 patients who had abdominal pain with ascites, 6 developed the symptom on day 6 of fever and one on day 8, and all responded well to antibiotic treatment, although one had a seizure. Lower respiratory symptoms were found in 7 patients, of whom one had underlying asthma; all had infection-induced wheezing and 5/7 patients developed the symptom before the 5^th^ day of fever. Anemia (Hct <30%) was the most common complication, found in 25/354 (6.8%) patients. Three patients developed hemolytic anemia, two of whom had an underlying disease, one with glucose-6-phosphate dehydrogenase (G-6 PD) deficiency and the other with hemoglobin H disease, with the lowest hematocrits of 16% and 18%, respectively. The patient who had an unknown cause of hemolytic anemia also had acute kidney injury (AKI) with an initial blood urea nitrogen (BUN)/creatinine (Cr) level of 33/2.3 mg/dL; his BUN/Cr level decreased to 14/1.1 mg/dL on day 4 after beginning treatment. Fifteen patients had thrombocytopenia, 4 with <50,000 platelets/mm^3^ but none with active bleeding.

### Sensitivity, specificity, negative predictive value and positive predictive value of serology tests for typhoid fever

The mean day of fever when blood was drawn for the serology tests was 6.0±4.0 days. Of the 368 blood-culture confirmed patients, Typhi IgM and IgG tests were performed in 199 and 135 patients, respectively. The positive rate of both Typhi IgM and IgG tests did not increase when compared among the 3 periods of fever, before day 5, days 6–10, and after day 10; the three positive rates for the three periods were *S* ser. Typhi IgM (51.4%, 66.7%, 41.7%) and *S* ser. Typhi IgG (28.2%, 40.0%, 42.9%), respectively. The sensitivity, specificity, PPV, and NPV of the *S* ser. Typhi IgM tests were 56.3%, 74.1%, 31.4%, and 88.9%, respectively, and of the *S* ser. Typhi IgG tests were 33.3%, 50.5%, 7.7%, and 85.9%, respectively. Of the 126 cases who had both *S* ser. Typhi IgM and IgG rapid tests, 82 (65.1%) and 20 (15.9%) cases were positive to one of or both IgM and IgG, respectively (Table 3).

**Table 3:** Sensitivity, specificity, negative predictive value, and positive predictive value of *S* ser. Typhi IgM/IgG rapid test.

Test	Results				Sensitivity (%)	Specificity (%)	PPV(%)	NPV (%)
	TP	FN	FP	TN				
IgM rapid test	112	87	244	697	56.3	74.1	31.4	88.9
Typhoid IgG	45	90	536	548	33.3	50.0	7.7	85.9

TP, true positive; FN, false negative; FP, false positive; FN, false negative.

## Discussion

We found good clinical outcomes of most typhoid fever cases during the 2009–11 outbreak in Songkhla, Thailand, as most of the patients were treated within 2 weeks of the first appearance of fever and all of the *S* ser. Typhi strains isolated were susceptible to our standard first line antibiotics.

We found that both of the *S* ser. Typhi IgM/IgG tests had low sensitivity for diagnosing typhoid fever, with IgM only 56.3% and IgG only 33.3%. A previous study found higher sensitivity of both IgM (69.0%) and IgG (70.7%) [29]. The specificity of IgM in our study was similar to this same study (74.1% vs 79.3%), but we found a lower specificity of IgG (50.5% vs 76.9%) [29]. However, both our study and this previous study found a high NPV for both *S* ser. Typhi IgM and IgG tests, suggesting that it is safe for the physician to wait for blood culture results if the rapid test is negative.

The first outbreak lasted for 6 months and entered a period of remission between April and August 2010, which could be explained by the April to May summer school break in Thailand, along with the implementation of various measures to improve sanitation at the affected schools, including providing health education messages for communities and in schools, promoting hand washing for food handlers and students, avoiding foods and beverages from street vendors, cleaning and renovating the kitchens and toilets in the outbreak schools and nearby markets, closing the schools for 7 days during school renovation, encouraging the students to eat homemade lunches/beverages using their own plates and spoons, providing ciprofloxacin to all school food handlers for 14 days, and adding chlorine to the tap water to increase chlorine levels from 0.1 ppm to 0.5 ppm. After these measures were implemented, the number of typhoid cases decreased, and the epidemic seemed to go into remission in April; however, 3–4 months later, after the schools reopened, a second outbreak was underway, which had a greater number of cases than the first outbreak, and lasted for 11 months, after which the overall outbreak ended as typhoid levels returned to historical norms. During the second outbreak, the Ministry of Public Health in Songkhla expected that after control measures were implemented in the schools and in local markets, the outbreak would go into remission as in the first outbreak, so vaccinations were not given to the students, as the current typhoid vaccines are only moderately effective for short periods of time, are costly, and multiple doses are required [30], [31]. However, without accurate source identification as in our situation, typhoid vaccine implementation would be an important tool to help control the outbreak [32]. We found that PFGE profiles from the 52 tested samples had the same pattern, indicating that the *S* ser. Typhi strains isolated from the patients from different schools were likely to have come from the same source. A large outbreak in Nepal lasted only 8 weeks after the source of the outbreak was found in the water supply, which suggests that isolating the source of *S* ser. Typhi quickly is an important factor in controlling such outbreaks [23].

We found that all of the *S* ser. Typhi strains isolated were susceptible to all first line antibiotics, including ciprofloxacin and ceftriaxone, as all of our patients responded well to these antibiotics and none had treatment failure or relapse [22], [33]. According to the Thai health policy which requires patients to access services in their local health district, there were no patients who suffered a second episode of typhoid fever during the outbreak, which indicates that there were no typhoid relapses or treatment failures, although a few patients might have moved to another province.

Although ciprofloxacin is recommended as the first line antibiotic for the treatment of typhoid and was successfully used in our situation, other studies have noted a high resistance rate to first line antibiotics; for example, studies from the United States, Cambodia and Vietnam found multidrug resistant *S* ser. Typhi in 13%, 58%, and 85% of cases, respectively [24]–[26]. Monitoring the current resistance in typhoid endemic areas of *S* ser. Typhi susceptibility is important to ensure an appropriate antibiotic is used when an outbreak occurs.

We found, as in previous studies, that cough or wheezing was common in typhoid fever patients [10], [34]. Acute hemolysis can occur in patients who have thalassemia or G-6PD deficiency [34]. We found that one-third of our patients had a dry cough without rhinorrhea, similar to a previous study which found cough in 39% of the typhoid fever cases [10]. In general, typhoid complications such as abdominal pain or pneumonitis will develop in the second week in patients who do not receive an appropriate antibiotic [2], [35]. However, most of patients in our study who had abdominal pain with ascites developed symptoms by the end of the first week of illness. In addition, most of patients who had dyspnea developed the symptom within the first week of illness, but all responded well to antibiotics and bronchodilator.

In summary, there were 2 related outbreaks of typhoid fever in Songkhla during 2009–2011, with a 5-month gap between them. Most of the patients were diagnosed at an early stage and treated with a good outcome. Patients who were treated within the first five days of fever had no typhoid-related GI complications. The *S* ser. Typhi IgM and IgG tests (SD Bioline) had low sensitivity for diagnosing typhoid fever in Thai children.

## Supporting Information

Data S1 **Typhoid Outbreak in Songkhla, Thailand 2009-2011.**
(XLS)
